# Supplementary material for: Optimising Meropenem and Piperacillin Dosing in Patients Undergoing Extracorporeal Membrane Oxygenation Without Renal Dysfunction (MEPIMEX)
Source: Antibiotics (Basel). 2025 Sep 17;14(9):939. doi: 10.3390/antibiotics14090939 (PMC12466471; doi:10.3390/antibiotics14090939)
Supplement: Supplementary file 1 [file antibiotics-14-00939-s001.zip › antibiotics-3854121-supplementary.pdf]

Table S1. ECMO characteristics of the recruited patients.

Patient ID	Drug	Indication	ECMO duration	ECMO mode	Oxygenator membrane	Bloodpump
1	Piperacillin	ARDS	75	VV	Maquet Rotaflow	HLS
8	Piperacillin	ARDS	123	VV	Maquet Rotaflow	PLS
13	Piperacillin	ARDS	55	VV	CardioHelp	HLS
18	Piperacillin	ARDS	25	VV	Maquet Rotaflow	PLS
24	Meropenem	ARDS	21	VV	CardioHelp	HLS
27	Piperacillin	ARDS	45	VV	CardioHelp	HLS
35	Meropenem	ARDS	63	VV	CentriMag	HLS
43	Piperacillin	ARDS	49	VV	Maquet Rotaflow	PLS
48	Meropenem	ARDS	6	VV	Maquet Rotaflow	PLS
52	Meropenem	ARDS	40	VV	CardioHelp	HLS
55	Piperacillin	ARDS	40	VV	CardioHelp	HLS
60	Meropenem	ARDS	43	VV	Maquet Rotaflow	PLS
64	Piperacillin	ARDS	56	VV	Maquet Rotaflow	PLS
67	Piperacillin	ARDS	29	VV	CardioHelp	HLS
70	Piperacillin	ARDS	21	VV	CardioHelp	HLS
72	Piperacillin	ARDS	23	VV	CardioHelp	HLS
111	Meropenem	Acute myocardial infarction	11	VA	CardioHelp	HLS
186	Meropenem	Acute myocardial infarction	13	VA	CardioHelp	HLS

ARDS: Acute respiratory distress syndrome; VV: Veno-venous; VA: Veno-arterial.

Table S2. Clinical, analytical, and infectious characteristics and pharmacokinetic and pharmacodynamic parameters by samples included in the study.

	Control group	ECMO group	P-value
**Meropenem**			
Number of samples	53 (67.1)	26 (32.9)	
Clinical parameters			
MV	30 (56.6)	26 (100.0)	**< 0.001**
Post-surgical drainage	21 (39.6)	10 (38.5)	0.921
Drainage volume (mL/day)	413.5 (837.2)	148.0 (216.5)	0.333
Fluid balance (mL/day)	-16.4 (1601.7)	847.7 (1512.3)	**0.027**
Vasopressive therapy	15 (28.3)	11 (42.3)	0.213
Analytical parameters			
Serum creatinine (mmol/L)	0.67 (0.26)	0.49 (0.20)	**0.003**
eGFR, (mL/min/1.73 m^2)			
60-89	16 (30.2)	0	**<0.003**
90-119 (female)/129 (male)	26 (49.1)	14 (53.8)	

≥120 (female)/130 (male)	11 (20.8)	12 (46.2)	
Albumin (g/L)	29.2 (4.9)	25.9 (3.0)	**0.003**
Leukocytes (10^3/mL)	15.2 (19.9)	16.8 (9.3)	0.697
Infection parameters			
Type of treatment			
Empirical	38 (71.7)	11 (42.3)	**0.011**
Directed	15 (28.3)	15 (57.7)	
Type of infection			
Respiratory tract	21 (39.6)	19 (73.1)	**0.002**
Primary bacteremia	4 (7.5)	6 (23.1)	
Skin and soft tissue	5 (9.4)	0 (0)	
Intraabdominal	7 (13.2)	0 (0)	
Unknown	12 (22.6)	1 (3.8)	
Isolated microorganisms			
P. aeruginosa	6 (11.3)	5 (19.2)	
E. coli	6 (11.3)	2 (7.7)	
K. pneumonia	4 (7.5)	6 (23.1)	0.404
Other	10 (18.9)	2 (7.7)	
Unknown	27 (50.9)	11 (42.3)	
Pharmacokinetic and pharmacodynamic parameters			
fC_{ss} (mg/L)	21.9 (19.5)	9.4 (5.8)	**<0.001**
Dose normalised fC_{ss} (mg/L)	7.3 (6.5)	3.1 (1.9)	**<0.001**
CL_u (L/h)	2.14 (0.97)	2.76 (0.61)	**0.001**
fAUC (mg·h/L)	526.7 (468.8)	226.6 (138.8)	**<0.001**
fC_{ss}/MIC	17.5 (43.9)	5.3 (3.3)	0.163
**Piperacillin**			
Number of samples	100 (69.9)	43 (30.1)	
Clinical parameters			
MV	55 (55.0)	41 (95.3)	**< 0.001**
Post-surgical drainage	26 (26.0)	26 (25.6)	0.958
Drainage volume (mL/day)	240.8 (355.5)	240.5 (428.4)	0.998
Fluid balance (mL/day)	76.9 (1254.2)	73.4 (1262.5)	0.988
Vasopressive therapy	21 (21.0)	18 (41.9)	**0.010**
Analytical parameters			
Serum creatinine (mmol/L)	0.71 (0.21)	0.52 (0.24)	**<0.001**
eGFR, (mL/min/1.73 m^2)			
60-89	35 (35.0)	3 (7.0)	
90-119 (female)/129 (male)	54 (54.0)	20 (46.5)	**<0.001**
≥120 (female)/130 (male)	11 (11)	20 (46.5)	
Albumin (g/L)	29.4 (4.6)	27.5 (5.7)	**0.041**
Leukocytes (10^3/mL)	12.0 (5.7)	12.4 (4.7)	0.751
Infection parameters			
Type of treatment			
Empirical	70 (70.0)	22 (51.2)	**0.031**
Directed	30 (30.0)	21 (48.8)	
Type of infection			
Respiratory tract	80 (80.0)	39 (90.7)	
Primary bacteremia	3 (3.0)	4 (9.3)	0.071
Skin and soft tissue	7 (7.0)	0 (0)	
Intraabdominal	5 (5.0)	0 (0)	

Unknown	4 (4.0)	0 (0)	
Isolated microorganisms			
P. aeruginosa	18 (18.0)	9 (20.9)	
E. coli	6 (6.0)	3 (7.0)	
K. pneumonia	5 (5.0)	4 (9.3)	0.107
Other	20 (20.0)	5 (11.6)	
Unknown	51 (51.0)	22 (51.2)	
Pharmacokinetic and pharmacodynamic parameters			
fC_{ss} (mg/L)	32.1 (21.8)	23.6 (14.8)	**0.008**
Dose normalised fC_{ss} (mg/L)	2.7 (1.8)	1.8 (1.2)	**0.003**
CL_u (L/h)	2.95 (0.65)	3.30 (0.49)	**0.001**
*f*AUC (mg·h/L)	771.6 (523.7)	566.8 (356.2)	**0.008**
fC_{ss}/MIC	2.9 (2.9)	3.5 (5.3)	0.351

eGFR = estimated glomerular filtration rate (mL/min/1.73 m^2). Estimated creatinine clearance was calculated using CKD-EPI formula. ECMO = Extracorporeal membrane oxygenation. fC_{ss} = free plasma concentration at steady state. MV = mechanical ventilation. MIC = minimum inhibitory concentration. SD = standard deviation. *categorical variables expressed as n (%) and quantitative variables as mean (SD).
